# The ‘shades of grey’ in research integrity—Researchers admit to questionable research practices that they do not perceive to be serious

**DOI:** 10.1371/journal.pone.0339056

**Published:** 2026-01-12

**Authors:** Marta Entradas, Yan Feng, Inês Carneiro e Sousa

**Affiliations:** 1 Cies-Center for Research and Studies in Sociology, Iscte-Instituto Universitário de Lisboa (Iscte-IUL), Lisbon, Portugal; 2 Department of Psychological and Behavioural Science, London School of Economics and Political Science, London, United Kingdom; Institute of Medical Biochemistry Leopoldo de Meis (IBqM) - Federal University of Rio de Janeiro (UFRJ), BRAZIL

## Abstract

Research misconduct practices like fabrication, falsification and plagiarism (FFP) are serious deviations from good research conduct, which have attracted attention in the literature due to the damage they can bring to science and society. However, less is known about the grey zone of researchers’ behaviours that deviate from responsible research conduct but do not fall under serious research misconduct practices. These are known as questionable research practices (QRPs), and they are believed to pose a no less serious threat to research integrity and science. Despite increasing research on the topic, the extent of the problem in different research fields and contexts is unknown. Using a sample of researchers working in Portuguese universities in six main fields of research (n = 1573), we report on QRPs that researchers admit to and how serious they perceive them to be, and on predictors of engagement in QRPs. We find that QRPs are widespread across all fields of research and seniority levels. Yet, younger, more prolific researchers, and those dismissing the seriousness of QRPs admitted to more QRPs. This suggests that some groups are at higher risk of misconduct and that there is a need for studying the motivations behind more susceptible groups to engage in QRPs.

## Introduction

In an ideal world, science is good and scientists are competent people with good intentions. However, science is not always ‘pure’ and the way it is conducted can deviate from the values that guide good scientific work, with consequences to science and society. The problem of misconduct has gained growing interest from scholars to study integrity in science [1,2], but also from institutions that associate excellence in research with ethics and integrity [3].

Research integrity has often been studied by examining scientists’ breaches of integrity and infringements [4–8], highlighting various practice-based problems that occur in science. Whether misconduct in science has expanded in recent years, we cannot say. However, there have been estimates of an increasing, widespread practice among researchers in different fields of research, as suggested by the increasing number of studies [9–11]. Often associated with the worldwide spread of a “publish or perish” culture and pressure on researchers to publish results quickly and in specific scientific outlets [9,12,13], research integrity has become a preoccupation among all fields of research.

Yet, preoccupations with integrity in science are not new. Allusion to the concept has been made by Charles Babbage (1791–1871) in his ‘Reflections on the Decline of Science in England, and on Some of its Causes’ (1830), where he describes four types of scientifically dishonest behaviour, which are still relevant today: hoaxing, forging, trimming, and cooking [14]. Later, Robert Merton [15] showed similar preoccupations when he developed the ‘Mertonian norms’ to describe the scientific ethos. Merton posited four norms to describe the ideal scientific community in terms of institutional values that are internalized by the scientist, expressed as ‘Communism’ (also referred to as communalism) meaning common ownership of scientific discoveries and the need for scientists to publicly share their findings; ‘Universalism’ encompassing the idea that anyone can do science, regardless of race, nationality, gender or any other differences, and all should be scrutinized equally; ‘disinterestedness’ as scientists should work only for the benefit of science; and ‘organized scepticism’ meaning that scientific work should be conditional on assessments of its scientific contribution, objectivity and rigor.

Despite the terminology lacking uniformity (the terms ‘misconduct’, ‘research integrity’ and ‘scientific integrity’ are often used interchangeably) [16,17], ‘research integrity’ has become commonly accepted in recent years as an umbrella term to promote good research practices [18]. The 1992 Responsible Science report from the U.S. National Academies of Sciences, Engineering, and Medicine distinguished ‘*misconduct’,* which are serious breaches of research integrity such as falsification, fabrication and plagiarism (FFP), from *questionable research practices (QRP)*, which are less flagrant, more subtle behaviours of potential misconduct such as, for example, selective reporting of results, falsely attributing authorship, not reporting conflicts of interest, or data management issues [19]. Our study focuses specifically on questionable research practices.

Contrary to FFP that are perceived as fundamentally negative, QRPs often fall into an amorphous area of acceptable, responsible research practices and unacceptable, irresponsible research practices, and could be seen as the grey zone in a spectrum of black and white; these are more subtle research behaviours, difficult to identity and even ignored by researchers or stakeholders [20,21]. Yet, despite being no universal definition of QRP [10], there is consensus that such conduct is irresponsible, involving misrepresentation, inaccuracy, or bias [22] and their damage is believed to exceed others [23]. The 2017 report from the NASEM recommends stopping calling these practices “questionable” because they have, in fact, been found to be detrimental to science and clear violations of the fundamental tenets of research, and should be treated as misconduct. Here, we use the term questionable research practices (QRPs hereafter) in keeping with the more common usage.

Regardless of the term, there are so many different forms of breaches to integrity that investigating them has become complex [24]. Studies have used different QRPs and methodologies, making it difficult to compare and assess trends over time. Also, few studies have presented models to predict this behaviour. Despite this, QRPs seem more prevalent than serious misconduct practices, and widespread among researchers. According to a meta-analysis of surveys on research integrity (N = 18), an average of 2% of scientists admitted to having committed at least one serious form of misconduct (fabricated, falsified or modified data or results) – and up to 34% scientists admitted to other QRPs [25]. Similarly, Xie et al. [10], analysing studies from 2011 to 2020, show that the estimates of researchers’ self-reported practices involving at least one FFP were close to 3% and to 13% for QPRs, while around 16% said they had witnessed others commit at least one form of misconduct, or a QRP (40%).

### QRPs across individual factors and research questions

The available research suggests that breaches in science are common among researchers in many countries and areas of research, as shown by studies in Spain [26], the Netherlands [27], Denmark [28], Norway [29], Italy [e.g.,^30^], the USA and Europe (including UK, Norway, Iceland and Switzerland) [8], Pakistan [31], India [32] and Brazil [33]. For example, in a survey of 1353 scientists from different fields of research attendees of the World Conference on Research Integrity (WCRI), Bouter and colleagues [34] listed the top 5 of the 60 misbehaviours reported. These included in decreasing order, ‘selectively cite to enhance your own findings or convictions’, ‘insufficiently supervise or mentor junior co-workers’; ‘not publish a valid negative study’; ‘demand or accept an authorship for which one does qualify’; ‘selectively cite to please editors, reviewers or colleagues’. In a survey among Dutch researchers [27], about 52% admitted to at least one QRP in their academic life (out of the 11 practices listed), and in a study among Norwegian researchers about 40% admitted to at least one QRPs (out of nine listed) in the three years prior to the study [29].

While findings are not conclusive and patterns are difficult to trace, the literature points to variations in admitted to by scientists in different research fields, levels of seniority and socio-demographic characteristics [e.g., 8,10,35]. Misconduct has been more frequently found among male scientists than female, younger, less senior researchers [31,36,37]. But studies of QRPs have been contradictory. For example, Martinson et al. (2005) found higher admission rates of QRPs among older researchers; Agnoli et al. [30], among senior Italian researchers than among junior/middle researchers. There are also studies showing that the occurrence of misconduct seems to have no relation to the individual’s seniority level, suggesting that it can happen across the whole career spectrum [36].

As such, despite it being often assumed that younger researchers and those in early professional stage would be more likely to show questionable behaviours due to the pressure to publish, instability and scarcity of research professional positions [2], lack of experience and poor supervision [28], such arguments do not seem to be supported by the available research or might hold only in specific contexts (e.g., research environment, competitiveness of the institution, internationalisation, promotions, etc). This idea is reinforced by Allum et al. [8], who found that, in Europe, the frequency with which mid– or senior–level researchers admitted to QRPs was lower than that of junior researchers, while in the US, junior researchers admitted to fewer QRPs than middle-level or senior researchers.

Similarly, no patterns have been found regarding research field. Bouter and colleagues [34] show that misbehaviours are more frequent in the social sciences than in other fields; for instance, QPR ‘selectively cite or cite your own work to improve citation metrics’ was more common in the biomedical sciences. In contrast, Allum et al. [8] found that researchers from natural and medical sciences admitted to more QRPs than social sciences and humanities researchers, and Ravn and Sørensen [37] show similarities across fields in committing certain practices across fields.

As previous studies point to mixed results or no significant differences among most of those factors, we do not hypothesise about tendencies in QRPs (across gender, age or seniority) among our community of researchers, also because of the particularities of this community. Embedded in a national research context of precarious ‘careers’ fed by a system of continuous fellowships (with few stable contracts) provided by the government, research careers in Portugal have been insecure and uncertain. There is a high prevalence of researchers in junior positions, regardless of age, with researchers staying for years if not decades in temporary contracts, and stagnation in promotions [e.g., 38,39]. We asked the following research question:

*RQ1: What QRPs do scientists working in Portuguese universities admit to committing and how does engaging in QRPs vary across individual characteristics?*

In addition to socio-demographics, we investigated the effects of two other factors not yet examined in the literature as possible predictors of questionable behaviour – the academic performance of a researcher as given by the number of publications and the perceived seriousness of misconduct practices. Maggio and colleagues [40] found that the number of publications in health education professions had a significant positive association with misconduct. Moreover, seen as an indicator of ‘visibility’ and performance, those more published scientists, likely involved in large projects and collaborations, could also be more persuaded to engage in QRPs, or simply want to maintain or grow their ‘status’ [2]. This expectation can, however, be challenged by the fact that those more productive researchers could also be more aware of guidelines and ethical procedures in the international environment they move in, thus behaving to avoid them. We would expect the number of publications to be associated with admitting to QRPs *(H1)*.

In addition, recent evidence points to differing perceptions of what QRPs are more severe and prevalent, and these are influenced by disciplinary traditions, nature of research and collaborative practices [38]. For example, Sacco, Bruton, and Brown [21] show that behaviours that align most closely with the standard definition of research misconduct (i.e., FFP) were regarded as highly unethical and indefensible. Conversely, behaviours aligning with the definitions of QRPs were seen as being more ethically defensible than others.

This could suggest a relationship between the perceived seriousness of QRPs and whether a researcher admits to them. The Social Cognitive Theory [SCT; 41] proposes that individuals develop their morality based on value judgements (i.e., standards of right and wrong) that guide moral conduct. Thus, researchers who believe that QRPs are a serious violation of scientific integrity standards may be less willing to engage in behaviours of misconduct. We asked the following RQ and tested the hypothesis:

*RQ2: How serious do scientists working in Portuguese universities see QRPs to be and how do those views vary across individual characteristics?*

*H1: Perceived seriousness of QRPs is negatively associated with engagement in QRPs.*

Finally, we investigated which of the individual factors under study were good predictors of admitting to QRPs. We investigated individual characteristics already brought up in different studies as potential individual factors influencing misconduct, and we brought up new factors, which we think might influence scientists’ behaviour. To the best of our knowledge, research integrity practices among this community have not been studied yet, nor has a predictive model on the likelihood of engaging in QRPs been tested. We asked:

*RQ3: What factors are associated with engaging in QRPs?*

## Methods

### Sample design and data collection

We built our sampling frame from articles published in the Web of Science by researchers working in Portuguese universities. The articles were selected using as search criteria ‘country (Portugal)’ and ‘year (2010-2023)’. We acknowledge that this does not necessarily correspond to the population, as there might be researchers in Portuguese universities who have not published articles in journals listed in the Web of Science in this period. After cleaning for incomplete records, duplications of records, authors, and emails, we obtained a sampling frame of n = 29,879 contacts of active researchers.

Due to resource limitations for data collection, we randomly selected n = 10,018 contacts as our sample. We first contacted respondents to advertise the study and to verify email addresses. From this, we deleted those contacts that informed us they did not want to participate or were no longer ‘active’ (e.g., left academia) and bounced emails (did not reach the respondent), reaching a final sample of n = 9,062 researchers.

Each researcher received a unique link, meaning that the questionnaire could only be completed by the link’s receiver. Questionnaires completed to less than 70% were discarded. The data were collected between 29 November 2023 and 8 February 2024, using Qualtrics software. Four reminders were sent during this period. No personal data were collected. Electronic consent was requested for participation: participants were informed at the beginning of the survey about the nature of the study, that participation was voluntary, no risks were foreseen, and confidentiality was assured (responses were anonymous with no possibility of identifying the participating institution or participants). Ethical approval was granted by Iscte Ethics Committee Review (Ref: 121/2023).

### Measures and questionnaire

To contribute to the harmonisation of scientific integrity measures, most of the items used in this study were adapted from the project SOPs4RI (Standard Operating Procedures for Research Integrity) [42], and from other previous work [e.g., 4,9,21]. The questionnaire was developed in English and translated into Portuguese, and back translated into English following a translation back-translation process [43]. Participants could answer in Portuguese or in English. Questionnaires were piloted among a few researchers and final adjustments were made. Twelve items on QPRs were used in the questionnaire, as shown in Table 1, and we asked researchers how frequently they have engaged in any of those practices and how serious they saw them.

**Table 1:** Questionable research practices listed in the survey.

Questionable research practices
I failed to cite publications that contradict my beliefs
I did not conduct a thorough literature review
I chose not to report my own findings if they contradicted my theories
I used a researcher’s idea without giving credit
I failed to disclose conflicts of interest
I included authors who had not contributed sufficiently
I inadequately supervised a junior co-worker
I carried out research without ethical approval
I cited papers without consulting the primary source
I developed hypotheses after seeing the results
I cited scientifically irrelevant publications out of dependence or friendship
I cited publications only because they were already visible in the scientific community

#### Dependent variable.

*Engagement in QRPs.* This is a continuous variable computed from the sum of all reported QRPs (out of twelve) (‘non-applicable’ answers were excluded from the count). The index ranges from 0 to 12, meaning that each respondent gets a numeric value representing the total number of practices he/she reported (M = 4.3, SD = 2.5; Median = 4). Those admitting to more QRPs rank higher in the index. Reliability analysis shows high internal consistency for the 12 items (Cronbach’s α = .71). Respondents were asked ‘how frequently have you engaged in any of the following practices?’ in a scale from ‘very often (4) to never (1). The term ‘questionable research practices’ was not mentioned in the survey to avoid passing the idea of wrongdoing and influencing responses.

This variable is used as a dependent variable in the ANOVA and Bonferroni tests to investigate differences among and between groups for variables age, level of seniority, field of research, number of publications; and in the regression analysis to predict a researcher’s likelihood of engaging in QRPs.

### Independent variables

*Perceived seriousness of QRPs.* We asked participants “how serious do you perceive the following activities to be” on a 4-point Likert scale ‘Not serious at all’ (1), ‘Not very serious’ (2), ‘Serious’ (3), ‘Very serious (4), and ‘Don’t know’ (5). We transformed this variable into an index (degree of seriousness) for purposes of ANOVA and regression analysis.

*Index of degree of seriousness.* We built this index from the 12 items of seriousness, creating a continuous variable that represents the degree of perceived seriousness in a ratio scale, obtained by computing the mean of all reported QRPs. The scale ranges from (1) to (4), meaning that each researcher gets a numeric value representing perceived seriousness, in which the highest point is 4 (high perceived seriousness) and the lowest point is 1 (low perceived seriousness). Reliability analysis shows a Cronbach’s Alpha = .79 for the 12 items (M = 3.27, SD = .38; Median = 3.3).

*Gender* was coded ‘female’ (1), ‘male’ (2), ‘other’ (3), ‘prefer not to say’ (4). Options (3) and (4) were treated as missing cases in the statistical analysis (they represented less than 1% in the sample).

*Age* was ordinally coded into four groups: ‘18-39’ yrs (1), ‘40-49’ (2), ‘50-59’ (3), ‘60 or more’ (4).

*Field of research* was given by six categories following the Frascati classification [44] into ‘Natural Sciences’ (1), ‘Engineering and Technology’ (2), ‘Medical and Health Sciences’ (3), ‘Agricultural and Veterinary Sciences’ (4), ‘Social Sciences’ (5), and the ‘Humanities’ (6).

*Seniority level* was originally an ordinary categorical variable (7 categories) recoded into three: ‘Junior’ (Assistant Professor + Junior researcher + PhD student) (1), ‘Mid-career’ (Associate Professor + Mid-career researcher) (2), ‘Senior’ (Full professor + Senior researcher) (3).

*Number of publications in the last 5 years* was used as a proxy indicator of a researcher’s performance. It is an ordinal variable coded ‘0 to 5 publications’ (1), ‘6 to 10 publications’ (2), ‘11 to 20 publications’ (3), ‘more than 20 publications’ (4) (M = .67, SD = .17518, p < .001).

### Statistical analysis

We used one-way ANOVAs to compare groups (S2 and S3 Tables) and pairwise Bonferroni post hoc tests to determine which groups differed significantly from each other, and use hierarchical regression models to investigate the effects of the independent variables on the likelihood of *engaging in QRPs* (dependent variable). The underlying rationale to add variables in two steps considered that we do not have theoretical assumptions to define what factors are more likely to show a significant improvement in the proportion of explained variance in our dependent variable. Model 1 considered demographic characteristics (gender and age), seniority level, and number of publications as potential determinants of engagement in QRPs, and Model 2, added ‘perceived seriousness’ (see Table 3 for results of regression analysis) to examine the separate and combined effects of variables.

**Table 3:** Regression analysis of ‘dependent variable ‘engagement in questionable research practices’ and individual factors ‘gender’, ‘age’, ‘seniority level’, ‘field of research’, ‘number of publications’ and ‘perceived seriousness of questionable research practices’ (n = 1272).

	Model 1			Model 2		
	*B*	*SE B*	*β*	*B*	*SE B*	*β*
*Step 1*						
Gender	0.14	0.13	0.03	−0.08	0.12	−0.02
Age	−0.30	0.07	−0.13***	−0.22	0.07	−.10***
Seniority level	0.16	0.10	0.05	0.18	0.09	0.06
Field of research	0.08	0.04	0.05	0.08	0.04	.05*
Number of publications (past 5 yrs)	0.30	0.06	.14***	0.36	0.06	.16***
*Step 2*						
Perceived seriousness of QRPs				−2.23	0.17	−.35***
(Constant)	3.73	0.32		11.00	0.62	
*R Square*	0.03			0.15		
*R*^*2*^ *change*	0.03			0.12		
*F* for change in *R*	8.37			180.06		

*** < 0.001; ** < 0.01; * < 0.05.

We analysed the effects of the predictors by comparing the standardised regression coefficients, and reported the adjusted R^2^, R^2^ Change, and F values, Unstandardised coefficients, and Standardised Beta values, and p values. We used an alpha level of.05 for all statistical significance. The model was significant and explained a considerable amount of the variance (15%). Analyses were conducted with SPSS v. 29.

## Results

We first describe the profile of the respondents. From the n = 9002 researchers contacted, we received n = 1573 responses, for a response rate of 17.4%. Approximately 54% are female researchers (46% male), the majority occupy junior positions (46%), whereas 18% are in senior positions, 36% are mid-career, working across all fields of research. This sample is demographically similar to the Portuguese scientific community for demographics gender, and seniority level, and fields of research [45], with a slight underrepresentation of the humanities. The detailed profile of the respondents is presented in S1 Table.

**Fig 1:**
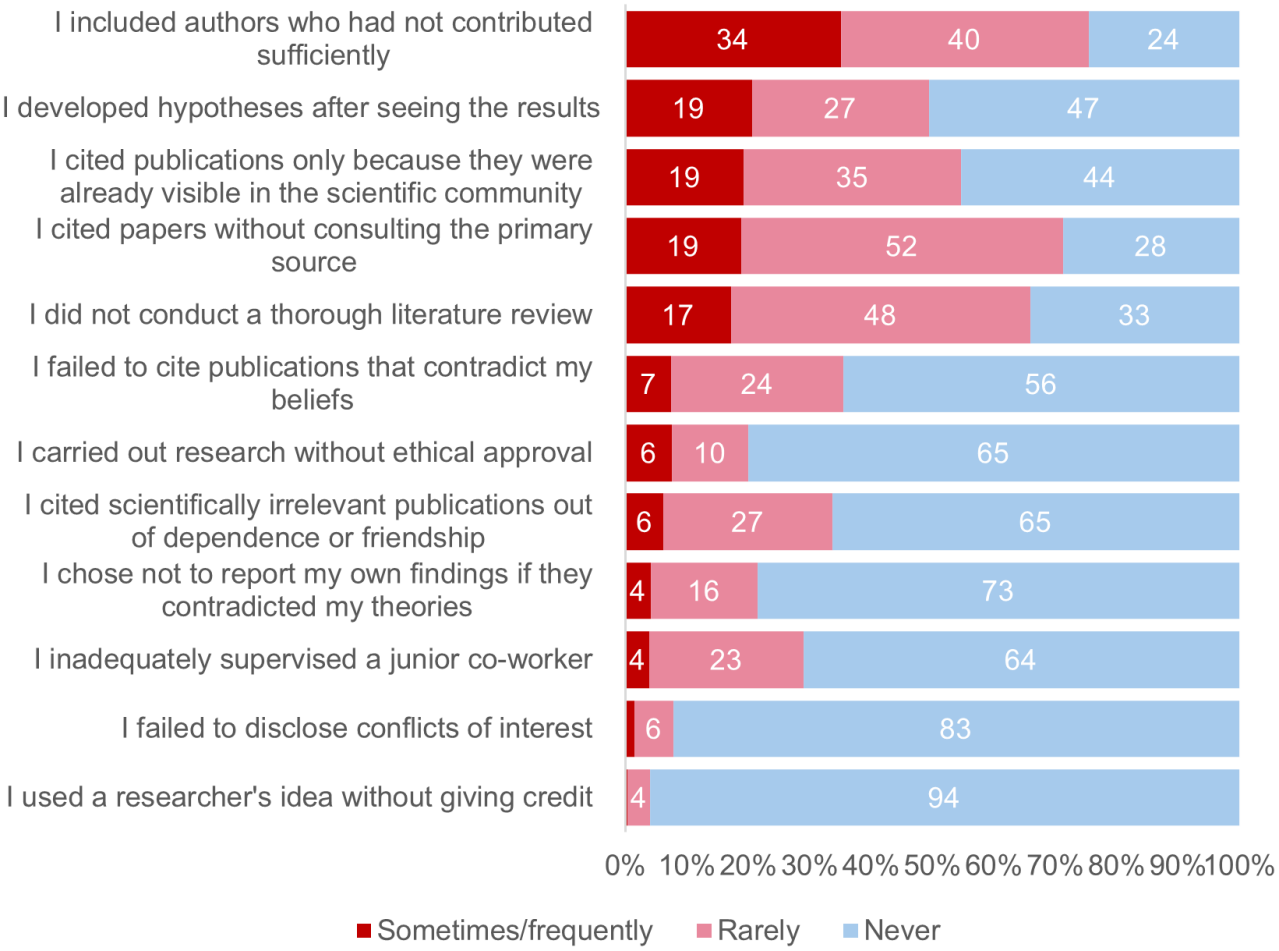
Percentage of researchers engaging in questionable research practices. The figure shows percentages for ‘never’, ‘rarely’, ‘sometimes/frequently’ in decreasing order (n = 1573).

To address RQ1, we analysed questionable practices reported by scientists and how they varied across individual characteristics. Out of twelve, the average researcher reported engaging in four QRPs (M = 4.3, SD = 2.5; Median = 4), with approximately 91% reporting one practice and 32% six or more (Table 2). The most reported practices by this community were “I included authors who had not contributed sufficiently”, “I cited papers without consulting the primary source” and “I did not conduct a thorough literature review”. In contrast, only a minority said they engaged in practices such as “I used a researcher’s idea without giving credit”, and “I failed to disclose conflicts of interest”. Fig 1 shows the percentage of researchers engaging in each practice, and Table 2 provides a more detailed picture of how QRPs are distributed across the sample. It should be noted, however, that while some practices were very common, a large part of the community has never engaged in many of the listed practices.

**Table 2:** Number and percentage of questionable research practices reported by researchers working in Portuguese universities (n = 1573).

Number of practices	Number of researchers	% of researchers reporting QRPs	Number of practices	% of researchers reporting QRPs
None	142	9	One	91
One practice	90	6	Up to two	85
Two practices	164	10	Up to three	75
Three practices	184	12	Up to four	63
Four practices	245	16	Up to five	48
Five Practices	245	16	Six or more	32
Six practices	204	13		
Above six practices	299	19		
**Total**	**1573**	**100**		

The number of QRPs reported varied among groups for number of publications (F(3, 1569) =6.793, p < .001) and age (F(3, 1395) = 3.296, p = .02), with those publishing more and younger being also more likely to admit to QRPs. Differences are not significant for gender, seniority level, and field of research. That is, QRPs are equally frequent among male and female researchers, junior and senior, and different field of research. Post-hoc tests for comparisons show that those more published researchers (> than 20 publications in the past five years) reported higher number of questionable practices than those publishing less – specifically, those publishing between 1–5 publications (M = .65, SD = .18, p = .002) and between 6–10 publications (M = .67, SD = .18, p < .001); yet age differences were not detected by the post hoc tests did not find single pairwise comparisons for age, meaning that the effects in the ANOVA were not large enough to overcome their stricter significance threshold. These differences are however captured in the regression analysis (see S2 Table for ANOVA results on reported practices).

### Perceived seriousness.

**Fig 2:**
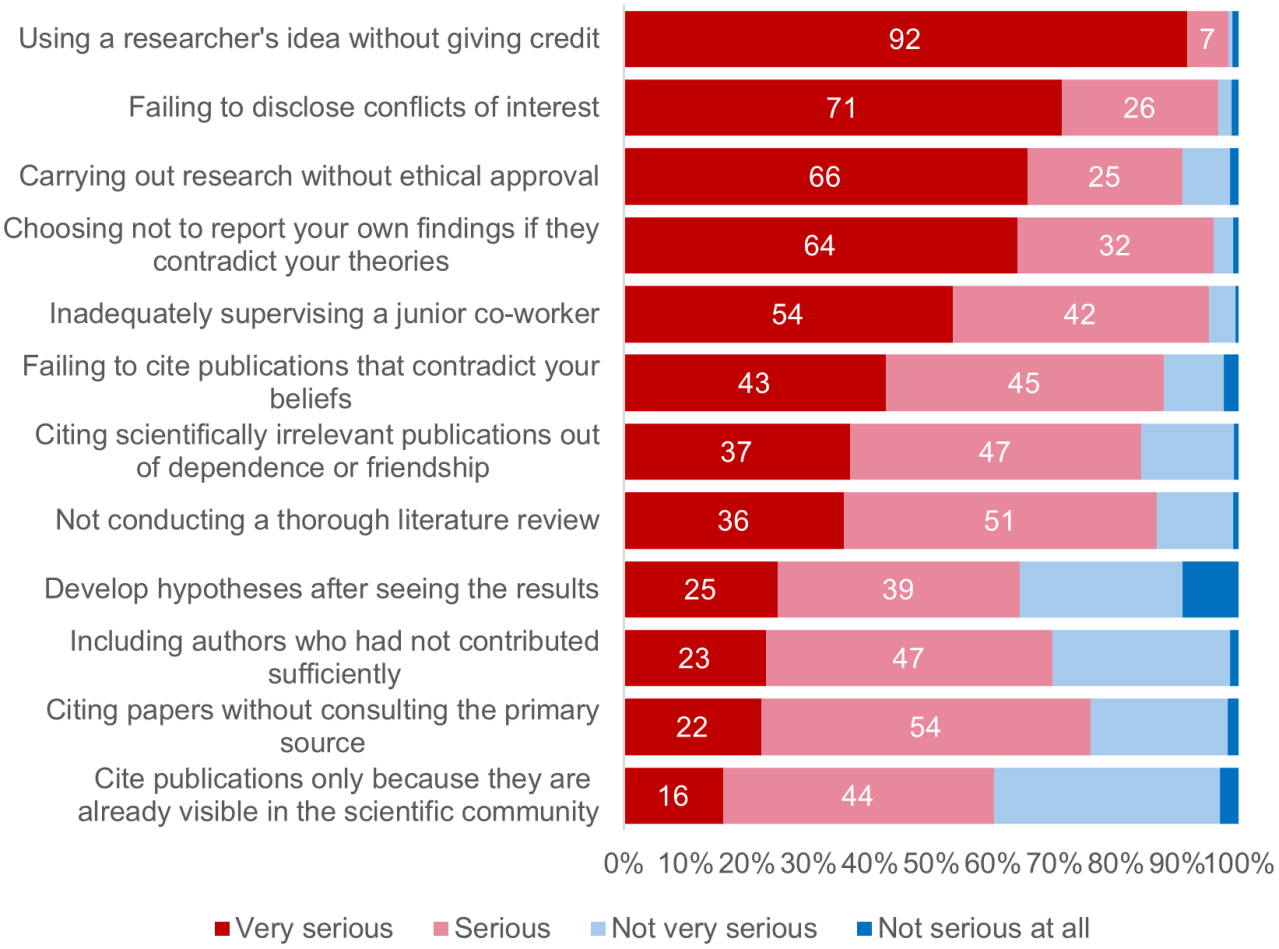
Perceived seriousness of each questionable research practice by researchers. The figure shows percentages for ‘very serious’, ‘serious’, ‘not very serious and ‘not serious at all’ (n = 1573).

We asked researchers how serious they perceived each practice to be (Fig 2) (RQ2). Most practices were seen as being serious, with the degree of perceived seriousness among researchers varying between 2.8 and 3.9 (in a range from 1 to 4 in the seriousness index) (see S3 Table for Means and SDs for each QRP). In decreasing order, practices seen as most serious were “using a researcher’s idea without giving credit”, “failing to disclose conflicts of interest” and “carrying out research without ethical approval”. In contrast, “citing publications only because they are already visible in the scientific community”, “including authors who had not contributed sufficiently”, and “developing hypotheses after seeing the results” were seen as not serious.

The ‘perceived seriousness’ of the listed QRPs varied with individual characteristics. QRPs were perceived to be more serious by female researchers (*p* < .001), older researchers – between 50–59 and older than 60 (*p* < .001), senior (p = 0.026), and more published researchers (p = .025). There was no significant relationship between perceived seriousness and fields of research; QRPs were perceived equally serious across fields of research (see S2 Table for ANOVA results for perceived seriousness).

### Predictors of engagement in QRPs.

To examine factors affecting engagement in QRPs (RQ2), we ran multiple regression analyses (Table 3). Regardless of the lack of variation between groups for some of the variables, as shown in the ANOVA and Bonferroni tests, relationships with the dependent variable might be affected when other variables are considered and together in the model.

When all conditions are controlled, Model 1 shows a significant relationship between ‘age’ and engagement in QPRs, and between ‘number of publications’ and engagement in QRPs, yet these variables explain only a small percentage of the variance in engagement in QRPs. Model 2 shows a better fit for the data, explaining a larger percentage of the variance in the level of engagement of QRPs (Table 3). Overall, in decreasing order of importance as shown by the effects (B), Model 2 shows a strong, significant relationship between ‘perceived seriousness’ and engagement in QRPs (B = −.353, *p* < .001), exerting by far the largest effect and explaining the largest variance, and confirming our H1. There is a significant relationship between ‘number of publications’ and engagement in QRPs (B = .164, p < .001), and between ‘age’ (B = −.095, *p* = .001) and ‘field of research (B = .052, p = .045) and engagement in QRPs, yet these are weak.

We note that ´research field’ becomes significant when ‘perceived seriousness’ is added in Model 2, suggesting that researchers in different fields might hold different views on the seriousness of these practices, and that the area of research might work as a moderating factor. This, however, needs further investigation.

## Discussion

Over the past decades, research integrity has become a research topic in itself and its focus expanded from more explicit and harmful forms of research misconduct to QRPs, with researchers examining their prevalence and specific factors [38,46]. Built on previous studies [e.g., 4,8,20,27], we investigated QRPs among researchers working in Portuguese universities. We reported first-time evidence of those practices among this community, and discuss here the main findings.

One main finding is that some of the studied questionable research practices were commonly admitted to by researchers, with about 92% reporting one practice and most between 4–6 practices on average. Most reported QRPs related to the writing of manuscripts, mostly to issues of co-authorship and citations, such as “including authors who had not contributed sufficiently”, “citing papers without consulting the primary source”, and “not conducting a thorough literature review”. To the contrary, practices such as “using a researcher’s idea without giving credit” and “failing to disclose conflicts of interest” were among the least mentioned. These findings corroborate others in studies that have used the same or similar items [e.g., 4,8]. They suggest that the QRPs in which this community engages are no different from those reported in other scientific cultures or countries. Rather, they seem widespread, pointing to a homogeneous pattern of QRPs to which researchers are more likely to engage in (and not). At the same time, these practices point to goals of speedy, quick reporting of results, and script of literature reviews, supporting the idea of existing pressure to publish [2]. Moreover, in our study, the percentage of researchers reporting one QRP is higher than in other studies, pointing to a culture where some of the studied practices seem to have become embedded.

Second, we found no significant differences in the number of QRPs reported by researchers working across the different research fields, levels of seniority, and between male and female researchers. This corroborates some studies and contradicts others that found differences across these factors. This inconsistent conclusion concerning the seniority level is well illustrated in the study of Allum et al. [8], which found that in the US, mid and late career researchers reported more QPRs than those in early career, while in Europe, the reverse was found, with the early researchers reporting more QRPs than their senior colleagues. This suggests that such differences might be more an indication of the varied contextual and institutional environments in which scientists work than individual characteristics themselves, as we shall develop further in this discussion.

More revealing characteristics of questionable behaviour in this community, however, are the age, the ‘performance’ of researchers (number of publications) and their views about how serious these behaviours are. The fact that more published researchers are more likely to engage in QRPs (20%) is not entirely surprising. These are often more established researchers, likely to be part of international networks and collaborations, possibly co-authoring articles with large teams, which might facilitate engagement in such practices. This is problematic: more productive researchers are also those more likely to contribute to the spread of detrimental practices and research.

As for the age differences, researchers aged between 18–39 reported more QRPs than older researchers (50–59). One possible explanation for these differences is that younger researchers, facing more pressure to perform and establish themselves in the field as independent researchers, might more easily disregard the consequences of these practices. As we show here, younger researchers were also less conscious of the seriousness of detrimental practices, whereas older researchers held stronger views on their significance. Additionally, the sense of integrity develops over the course of one’s life and experience in research, managing projects and supervising others’ work, which can significantly influence individual views on research integrity [47]. However, the possibility that older researchers, holding higher status, may be more influenced by social desirability, thus reporting lower engagement in these practices, cannot be ruled out.

Thirdly, researchers do not associate their misbehaviours with misconduct. The most reported practices were perceived as not serious by the large majority, and the least reported were seen as serious. For example, in our study, “using a researcher’s idea without giving credit” was identified as the most serious misbehaviour, with 94% of participants stating that they never engaged in it. These results support in part the idea of moral reasoning in SCT [41], in which individuals develop their morality based on value judgements (i.e., standards of right and wrong) that guide conduct. The more serious those practices are perceived, the less likely researchers are to admit to them, possibly to preserve their moral self-image, especially when the practices are perceived as very unethical. This reasoning might be more prominent among certain groups.

In this regard, we note the ambiguity in some of the items concerning their seriousness, which leads us to the idea that some practices are considered serious in a straightforward way, while others are perceived ambiguously. This is consistent with the results of Banks et al. [48], identifying situations where QRPs do not seem problematic (‘the good’) and others in which these practices represented a serious threat to scientific results (‘the ugly’). Ravn and Sørensen [37] also reported that, in very specific cases, QRPs in one field of research are considered good research practice in another.

Overall, taken together, these findings tell us something about how researchers behave and perceive research integrity, and more specifically about the Portuguese community. First, the practices more commonly reported might have become accepted and normalised among some researchers. This is supported by the fact that, as shown here, admitted QRPs are not seen as serious. Second, engagement in QRPs is more prominent among certain groups. In our study, these tend to be younger and more productive researchers. These groups are at higher risk of perpetuating QRPs and their normalisation. In cases where QRPs are concerned with authorship issues, it can also lead to raising profiles of some less prolific researchers at the expense of others, more prolific ones [15]. Future research should investigate more deeply the characteristics and motivations of these groups for engaging in more QRPs.

We have highlighted here the potential of factors that can predict research integrity. We showed that ‘age, ‘number of publications’, and ‘perceived seriousness’ are good predictors of engagement in QRPs, while factors such as ‘seniority’, ‘gender’ and field of research were not good predictors for this community. This suggests that (lack of) integrity in each community might reflect more about a culture than individual factors. This argument is supported by our model, which, although confirms the importance of several individual factors as predictors of engagement in QRPs, the total variance explained is low.

The results also suggest that this sense of common, normalised practices might be more prominent among this community, perhaps triggered by the environment that researchers work in, a hostile national context for research where funding for research is low and precarious positions are the norm. Future research should investigate other factors, such as the institutional environment (support of institutions, views and policies, training and integrity guidelines, etc) to explain further why researchers engage in QRPs.

## Conclusion

This study contributes to the research integrity literature by extending our understanding of individual differences in the adoption of QRPs, and what factors relate to them, and in particular to this community.

The community of researchers working in Portuguese universities showed a pattern of engagement in QRPs that aligns with some cultures and scientific communities and contrasts with others. Our data urge us to think about research integrity not as a feature of discipline, but rather as a profile arising from an ordered combination of individual and possibly contextual factors.

We show that perceived seriousness is a significant predictor of engagement in QRPs. This indicates the importance of researchers being conscientious about what research integrity entails. We cannot, however, expect that consciousness alone will lead to better practices. The institutional environment may either facilitate or impede the integrity of their researchers.

To better understand the driving factors behind misconduct, it might not be enough to consider individual factors; the context might play a role in supporting researchers [e.g., 49]. Future models should consider contextual factors, including indicators of the institutional setting and support, but also other individual factors such as the type of contract with the institution, awareness of misconduct, and integrity principles.

These findings call attention to the need for a broader discussion within scientific communities and institutions on what research integrity entails, and the importance of institutions in guiding researchers in their ethical principles – through for instance: (i) the integration of comprehensive training and education in research integrity that goes beyond the basic tenets of FFP and to make researchers more conscious of detrimental practices and their consequences, while reinforcing the need to carefully follow the stages of the research process and clarifying the rules for assigning authorship [cf. 50]; and (ii) the need to rethink the system for evaluating researchers’ performance, taking into account factors other than quantitative publication metrics, while promoting a culture of rigor, transparency, openness, and ethical responsibility in research [e.g., 51].

### Limitations

The largest limitation of this study is that it relied on asking people to admit wrongdoing. This can be susceptible to social desirability bias as participants tend to respond in ways that make them look good or that align with social norms, rather than truthfully reflecting their actual behaviour. It is possible that participants might have been hesitant to admit they do something wrong. Also, participants were asked in the same survey about how frequently they engaged in the listed practices and how serious they considered each one of them to be, which might reflect biased answers due to concerns about the consistency of answers (i.e., common method bias). It is then possible that the real engagement in misconduct is higher than that reported by researchers. The fact that the surveys were anonymised might, however, decrease this bias. In addition, the fact that the survey might have attracted those more likely to respond to surveys or with an interest in the topic cannot be ruled out.

The survey included some behaviours that may be acceptable depending on the specifics of the case. Future studies should give the opportunity to respondents to clarify possible ambiguities or justify answers considering the variability of their fields of research, objects of study or source of data. Similarly, the concept of perceived seriousness remains insufficiently defined in the literature, as it is unclear which dimensions researchers consider when evaluating QRPs. For instance, do they assess seriousness based on the potential societal impact of the misconduct, the damage to the credibility and image of science, or the personal consequences they might face, such as reputational harm, legal or disciplinary sanctions? Future research can focus on a clearer understanding of the underlying evaluative criteria of perceived seriousness.

The response rate (17.4%), despite being low, is an acceptable rate for survey-based studies. Surveys of scientists often get lower response rates (many with 5–7%), with some exceptions. Despite our sample being representative of some of the socio-demographic characteristics of the Portuguese community of researchers, due to these possible biases, the data should be interpreted with care.

## Supporting information

S1 Table Profile of respondents. (DOCX)

S2 Table Statistical Analysis of Variance for QRPs’ admission using One-way ANOVA. We show the results of variance for the number of admitted practices among gender, age, seniority level, field research, number of publications and perceived seriousness of QRPs.
(DOCX)

S3 Table Statistical Analysis of Variance for perceived seriousness of QPRs using One-way ANOVA. We show the results of variance for the perceived seriousness of QPRs among gender, age, seniority level, field research, number of publications.
(DOCX)

S4 Table Mean and SD of perceived seriousness of QRPs. (DOCX)
