# Supplementary material for: The ‘shades of grey’ in research integrity—Researchers admit to questionable research practices that they do not perceive to be serious
Source: PLoS One. 2026 Jan 12;21(1):e0339056. doi: 10.1371/journal.pone.0339056 (PMC12795355; doi:10.1371/journal.pone.0339056)
Supplement: S1 Table — (DOCX) [file pone.0339056.s001.docx]

**S1Table.** Profile of the respondents (n=1573).

| **Variable** | **Category** | **Respondents** | |
| --- | --- | --- | --- |
|  |  | **n** | **%*** |
| Gender | Female | 741 | 54 |
|  | Male | 635 | 46 |
|  | **Total** | **1376** | **100** |
| Age | 18-39 | 308 | 22 |
|  | 40-49 | 433 | 31 |
|  | 50-59 | 415 | 30 |
|  | >=60 | 243 | 17 |
|  | **Total** | **1399** | **100** |
| Field of research | Natural Sciences | 373 | 24 |
|  | Engineering and Technology | 347 | 22 |
|  | Medical and Health Sciences | 316 | 20 |
|  | Agricultural and Veterinary Sciences | 103 | 7 |
|  | Social Sciences | 328 | 21 |
|  | Humanities | 104 | 7 |
|  | **Total** | **1571** | **100** |
| Level of seniority | Junior | 673 | 46 |
|  | Mid-career | 522 | 36 |
|  | Senior | 261 | 18 |
|  | **Total** | **1572** | **100** |
| Number of publications  (past 5yrs) | 1-5 | 336 | 21 |
|  | 6-10 | 404 | 26 |
|  | 11-20 | 437 | 28 |
|  | > 20 | 396 | 25 |
|  | **Total** | **1573** | **100** |

*Due to rounding up, percentages might not sum up to 100.
