# Supplementary material for: The ‘shades of grey’ in research integrity—Researchers admit to questionable research practices that they do not perceive to be serious
Source: PLoS One. 2026 Jan 12;21(1):e0339056. doi: 10.1371/journal.pone.0339056 (PMC12795355; doi:10.1371/journal.pone.0339056)
Supplement: S2 Table — We show the results of variance for the number of admitted practices among gender, age, seniority level, field research, number of publications and perceived seriousness of QRPs. (DOCX) [file pone.0339056.s002.docx]

**S2 Table.** Statistical Analysis of Variance for QRPs’ admission using One-way ANOVA. The table shows the results of variance for the number of admitted practices among gender, age, seniority level, field research, number of publications and perceived seriousness of QRPs (n=1573).

|  |  | **SS** | **df** | **MS** | **F** | **Sig.** |
| --- | --- | --- | --- | --- | --- | --- |
| Gender | Between Groups | 7.99 | 1.00 | 7.99 | 1.46 | 0.23 |
|  | Within Groups | 7543.98 | 1374.00 | 5.49 |  |  |
|  | Total | 7551.97 | 1375.00 |  |  |  |
| Age | Between Groups | 54.31 | 3.00 | 18.11 | 3.30 | 0.02 |
|  | Within Groups | 7663.38 | 1395.00 | 5.49 |  |  |
|  | Total | 7717.70 | 1398.00 |  |  |  |
| Seniority level | Between Groups | 11.61 | 2.00 | 5.81 | 0.94 | 0.39 |
|  | Within Groups | 9000.77 | 1453.00 | 6.20 |  |  |
|  | Total | 9012.39 | 1455.00 |  |  |  |
| Field of research | Between Groups | 60.24 | 5.00 | 12.05 | 1.95 | 0.08 |
|  | Within Groups | 9657.12 | 1565.00 | 6.17 |  |  |
|  | Total | 9717.37 | 1570.00 |  |  |  |
| Number of publications in the last 5 years | Between Groups | 124.82 | 3.00 | 41.61 | 6.79 | <.001 |
|  | Within Groups | 9609.15 | 1569.00 | 6.12 |  |  |
|  | Total | 9733.97 | 1572.00 |  |  |  |

Abbreviations: Sum of squares (SS); df (degrees of freedom); MS (Mean Square); F statistic (F) and (p (significance value)
