# Supplementary material for: The ‘shades of grey’ in research integrity—Researchers admit to questionable research practices that they do not perceive to be serious
Source: PLoS One. 2026 Jan 12;21(1):e0339056. doi: 10.1371/journal.pone.0339056 (PMC12795355; doi:10.1371/journal.pone.0339056)
Supplement: S3 Table — We show the results of variance for the perceived seriousness of QPRs among gender, age, seniority level, field research, number of publications. (DOCX) [file pone.0339056.s003.docx]

**S3 Table.** Statistical Analysis of Variance for perceived seriousness of QPRs using One-way ANOVA. We show the results of variance for the perceived seriousness of QPRs among gender, age, seniority level, field research, number of publications (n=1573).

|  |  | **SS** | **df** | **MS** | **F** | **Sig.** |
| --- | --- | --- | --- | --- | --- | --- |
| Gender | Between Groups | 2.90 | 1.00 | 2.90 | 21.32 | <.001 |
|  | Within Groups | 186.59 | 1373.00 | 0.14 |  |  |
|  | Total | 189.49 | 1374.00 |  |  |  |
| Age | Between Groups | 2.93 | 3.00 | 0.98 | 7.23 | <.001 |
|  | Within Groups | 188.21 | 1395.00 | 0.14 |  |  |
|  | Total | 191.14 | 1398.00 |  |  |  |
| Seniority level | Between Groups | 1.07 | 2.00 | 0.53 | 3.65 | 0.03 |
|  | Within Groups | 211.44 | 1450.00 | 0.15 |  |  |
|  | Total | 212.50 | 1452.00 |  |  |  |
| Field of research | Between Groups | 1.40 | 5.00 | 0.28 | 1.94 | 0.09 |
|  | Within Groups | 225.41 | 1562.00 | 0.14 |  |  |
|  | Total | 226.81 | 1567.00 |  |  |  |
| Number of publications in the last 5 years | Between Groups | 1.35 | 3.00 | 0.45 | 3.11 | 0.03 |
|  | Within Groups | 225.56 | 1566.00 | 0.14 |  |  |
|  | Total | 226.90 | 1569.00 |  |  |  |

Abbreviations: Sum of squares (SS); df (degrees of freedom); MS (Mean Square); F statistic (F) and (p (significance value)
