# Supplementary material for: The ‘shades of grey’ in research integrity—Researchers admit to questionable research practices that they do not perceive to be serious
Source: PLoS One. 2026 Jan 12;21(1):e0339056. doi: 10.1371/journal.pone.0339056 (PMC12795355; doi:10.1371/journal.pone.0339056)
Supplement: S4 Table — (DOCX) [file pone.0339056.s004.docx]

**S4 Table.** Mean and SD of perceived seriousness of QRPs (n=1573).

|  | **Mean** | **Std. Deviation** |
| --- | --- | --- |
| Failing to cite publications that contradict your beliefs | 3.3 | 0.7 |
| Not conducting a thorough literature review | 3.2 | 0.7 |
| Choosing not to report your own findings if they contradict your theories | 3.6 | 0.6 |
| Using a researcher's idea without giving credit | 3.9 | 0.4 |
| Failing to disclose conflicts of interest | 3.7 | 0.6 |
| Including authors who had not contributed sufficiently | 2.9 | 0.8 |
| Inadequately supervising a junior co-worker | 3.5 | 0.6 |
| Carrying out research without ethical approval | 3.6 | 0.7 |
| Citing papers without consulting the primary source | 3.0 | 0.7 |
| Develop hypotheses after seeing the results | 2.8 | 0.9 |
| Citing scientifically irrelevant publications out of dependence or friendship | 3.2 | 0.7 |
| Cite publications only because they are already visible in the scientific community | 2.7 | 0.8 |
